# Structure-based design of a SARS-CoV-2 Omicron-specific inhibitor

**DOI:** 10.1073/pnas.2300360120

**Published:** 2023-03-20

**Authors:** Kailu Yang, Chuchu Wang, Alex J. B. Kreutzberger, K. Ian White, Richard A. Pfuetzner, Luis Esquivies, Tomas Kirchhausen, Axel T. Brunger

**Affiliations:** ^a^HHMI, Stanford University, Stanford, CA 94305; ^b^Department of Molecular and Cellular Physiology, Stanford University, Stanford, CA 94305; ^c^Department of Neurology and Neurological Sciences, Stanford University, Stanford, CA 94305; ^d^Department of Structural Biology, Stanford University, Stanford, CA 94305; ^e^Department of Photon Science, Stanford University, Stanford, CA 94305; ^f^Program in Cellular and Molecular Medicine, Boston Children’s Hospital, Boston, MA 02115; ^g^Department of Pediatrics, Harvard Medical School, Boston, MA 02115; ^h^Department of Cell Biology, Harvard Medical School, Boston, MA 02115

**Keywords:** SARS-CoV-2, Omicron, membrane fusion, inhibitor, rational design

## Abstract

The Omicron variant of severe acute respiratory syndrome coronavirus 2 (SARS-CoV-2) introduced a relatively large number of mutations, including three mutations in the highly conserved heptad repeat 1 (HR1) region of the spike glycoprotein (S) critical for its membrane fusion activity. We show that one of these mutations, N969K induces a substantial displacement in the structure of the heptad repeat 2 (HR2) backbone in the HR1HR2 postfusion bundle. Due to this mutation, fusion-entry peptide inhibitors based on the Wuhan strain sequence are less efficacious. Here, we report an Omicron-specific peptide inhibitor designed based on the structure of the Omicron HR1HR2 postfusion bundle. Specifically, we inserted an additional residue in HR2 near the Omicron HR1 K969 residue to better accommodate the N969K mutation and relieve the distortion in the structure of the HR1HR2 postfusion bundle it introduced. The designed inhibitor recovers the loss of inhibition activity of the original longHR2_42 peptide with the Wuhan strain sequence against the Omicron variant in both a cell–cell fusion assay and a vesicular stomatitis virus (VSV)-SARS-CoV-2 chimera infection assay, suggesting that a similar approach could be used to combat future variants. From a mechanistic perspective, our work suggests the interactions in the extended region of HR2 may mediate the initial landing of HR2 onto HR1 during the transition of the S protein from the prehairpin intermediate to the postfusion state.

## Significance

The renewed threats from severe acute respiratory syndrome coronavirus 2 (SARS-CoV-2) by existing and emerging variants, including Omicron emerging sub-variants, require optimized development of vaccines and antiviral therapeutics. Vaccines and therapeutics targeting the critical viral spike glycoprotein (S) must accommodate the mutations in S. Even the highly conserved region that plays a critical role in membrane fusion exhibits up to three mutations in the HR1 region of Omicron variants. Here we show that one of these mutations causes a distortion in the structure of the SARS-CoV-2 postfusion structure, and we compensated for this structural change by structure-based design of an Omicron-specific peptide inhibitor that inhibits Omicron fusion and infection with low nanomolar activities.

Renewed threats from severe acute respiratory syndrome coronavirus 2 (SARS-CoV-2) are caused by a series of variants, including Omicron. The first strain of Omicron, B.1.1.529 or BA.1, was identified in South Africa in late 2021, and since then a variety of subvariants have evolved, including BA.1.1, BA.2, BA.2.12.1, BA.4, and BA.5 (1). These Omicron subvariants have an increased ability to escape neutralizing antibodies. For example, the BA.2.12.1, BA.4, and BA.5 subvariants show increased evasion compared to BA.2 of plasma-derived neutralizing antibodies from individuals who were triple-vaccinated or developed a BA.1 infection after vaccination (2), and the BA.2.75 variant shows enhanced evasion compared to BA.2 (3). New Omicron-specific vaccines are currently distributed although it has been suggested that they may not be much more effective than existing vaccines (4), and that they may be less efficacious against the most recent sub-variants BQ.1, BQ.1.1, and XBB.1.5 of Omicron (5–7). Moreover, infections in unvaccinated and immune-compromised individuals, breakthrough infections, and so-called long COVID are substantial public health concerns that warrant development of effective antiviral compounds in addition to existing vaccination regimens (8, 9).

To reduce the public health threat of Omicron and future emerging variants, we and others have proposed development of antivirals targeting the relatively conserved heptad repeat 1 and 2 (HR1 and HR2) regions of the spike glycoprotein (S) that drive membrane fusion by the formation of the HR1HR2 postfusion bundle (10–14). Although the ~130 residues of the HR1 and HR2 regions are conserved in all prior SARS-CoV-2 variants, a few mutations have reached fixation with Omicron in the HR1 region (Q954H and N969K for all Omicron subvariants, and, additionally, L981F for BA.1). Interestingly, the mutational load of the HR1 and HR2 regions is lower than that of certain other regions of the S protein; for the Omicron variants, the receptor binding domain (RBD) has 5 to 8 times more mutations per site than the HR1 and HR2 regions relative to the original Wuhan strain. The higher mutation frequency in the RBD domain is probably driven by evolutionary escape from neutralizing antibodies (15).

**Fig. 1.**
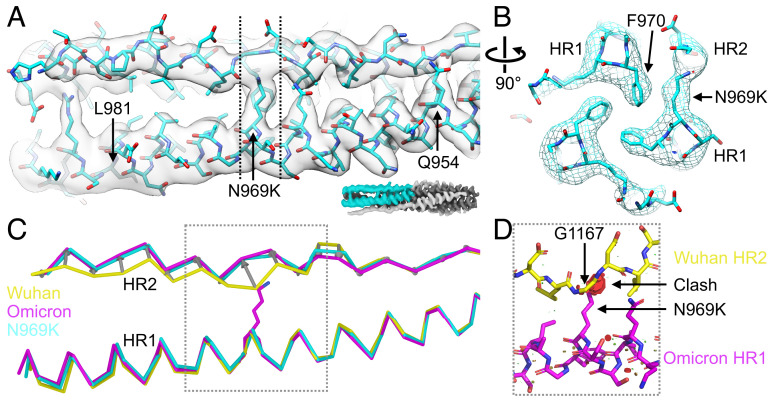
The N969K mutation alone induces the backbone shift observed in the Omicron HR1HR2 postfusion bundle. (*A* and *B*) Cryo-EM map and model of the HR1HR2 postfusion bundle with the N969K mutation (PDB ID 8fa1, EMDB ID 28947, this study). Cyan: carbon atoms of the N969K structure. Blue: nitrogen atoms. Red: oxygen atoms. Only one HR2 protomer and its two neighboring HR1 protomers are shown for clarity, and the displayed region of the bundle is colored cyan in the lower right inset. The dashed vertical lines indicate the slab that is displayed in panel *B*. (*C*) Superposition of the Cα ribbons of the postfusion bundle structures of the Wuhan strain (yellow, PDB ID 8czi, EMDB ID 27098, published in ref. 13), of the Omicron variant (magenta, PDB ID 7tik, EMDB ID 25912, published in ref. 14), and of the N969K mutant (cyan, PDB ID 8fa1, EMDB ID 28947, this study), viewed in the same orientation as that of panel *A*. Only one HR2 protomer and one neighboring HR1 protomer are shown for clarity. Gray arrows connecting the Cα atoms of Wuhan strain and Omicron variant HR2 backbones illustrate the backbone shift. (*D*) N969K in Omicron HR1 would clash with G1167 in the Wuhan strain conformation of HR2 when superimposing the two postfusion bundles. The carbon atoms are colored the same as that in panels *A* and *C*, i.e., yellow for Wuhan and magenta for Omicron, while the nitrogen and oxygen atoms are colored as blue and red, respectively. The clashes are shown as disks (red) whose radii are proportional to the severity of the clashes.

Previously, we reported an unmodified peptide with an extended sequence composed of 42 amino acids of the HR2 region, termed longHR2_42, that was designed to target these relatively more conserved HR1HR2 regions; this efficacious peptide inhibits infections by the original Wuhan strain and several variants of SARS-CoV-2 with an IC_50_ of ~1 nM (13). However, the inhibition activity of this peptide against the Omicron variant was around fourfold weaker in the vesicular stomatitis virus (VSV)-SARS-CoV-2 chimera infection assay and around tenfold weaker in the authentic SARS-CoV-2 infection assay. We hypothesized that this decreased inhibition activity is probably caused by the three mutations in the HR1 region—Q954H, N969K, and L981F—of Omicron BA.1. All three mutations are found at the HR1HR2 postfusion bundle interface and induce a backbone shift of HR2 (Fig. 1*C*) (14).

Here, we show that the N969K mutation alone is sufficient to displace the HR2 backbone in a similar manner to the Omicron BA.1 HR1HR2 postfusion bundle with all three Omicron mutations. Based on this finding, we designed an optimized inhibitor peptide, named 42G, to accommodate the distortion of the HR1HR2 postfusion bundle caused by the N969K mutation. The structure of the complex between the Omicron HR1 and the 42G peptide confirms our design—the 42G peptide forms a bulge to accommodate the N969K mutation. Functionally, the 42G peptide shows more potent Omicron-specific inhibition in both cell-based fusion and VSV-SARS-CoV-2 chimera infection assays compared to our original inhibitory peptide derived from the Wuhan strain.

## Results

### The N969K Mutation Alone Induces a Shift of HR2 in the Omicron HR1HR2 Postfusion Bundle.

Our structures of the HR1HR2 postfusion bundles of SARS-CoV-2 and its variants revealed a displacement of the HR2 backbone in the Omicron variant with the three mutations (N969K, Q954H, and L981F) in the HR1 region (PDB ID 7tik) (14). We thus determined the cryo-EM structure of the HR1HR2 bundle with the N969K mutation alone (Fig. 1*A* and *SI Appendix*, Fig. S1 and Table S1). The long sidechain of K969 is closely packed with F970 of a neighboring HR1 strand, which limits the possible rotamers of the K969 sidechain (Fig. 1*B*). The N969K mutant structure revealed a similar HR2 backbone displacement as observed in the Omicron HR1HR2 structure with all three mutations (Fig. 1*C*)—the average Cα atom rmsd for residues 1,162 to 1,170 is 0.7 Å between the N969K structure and the Omicron structure, 2.3 Å between the N969K structure and the Wuhan structure, and 2.4 Å between the Omicron variant structure and the Wuhan strain structure. This suggests that N969K alone is the major cause for the conformational change of HR2 in the postfusion bundle structure of Omicron. The comparison with the Omicron and Wuhan postfusion bundle structures illustrates structural rearrangement of HR2 to accommodate the long lysine sidechain at position 969 (Fig. 1*C*). The lysine would clash into the HR2 backbone if it were in the conformation of the Wuhan strain (Fig. 1*D*). Thus, the N969K mutation results in a major deflection of the HR2 backbone away from its Wuhan variant position to avoid steric clashes (Fig. 1*C*).

### Restoring the Interface between HR1 N969K and HR2.

**Fig. 2.**
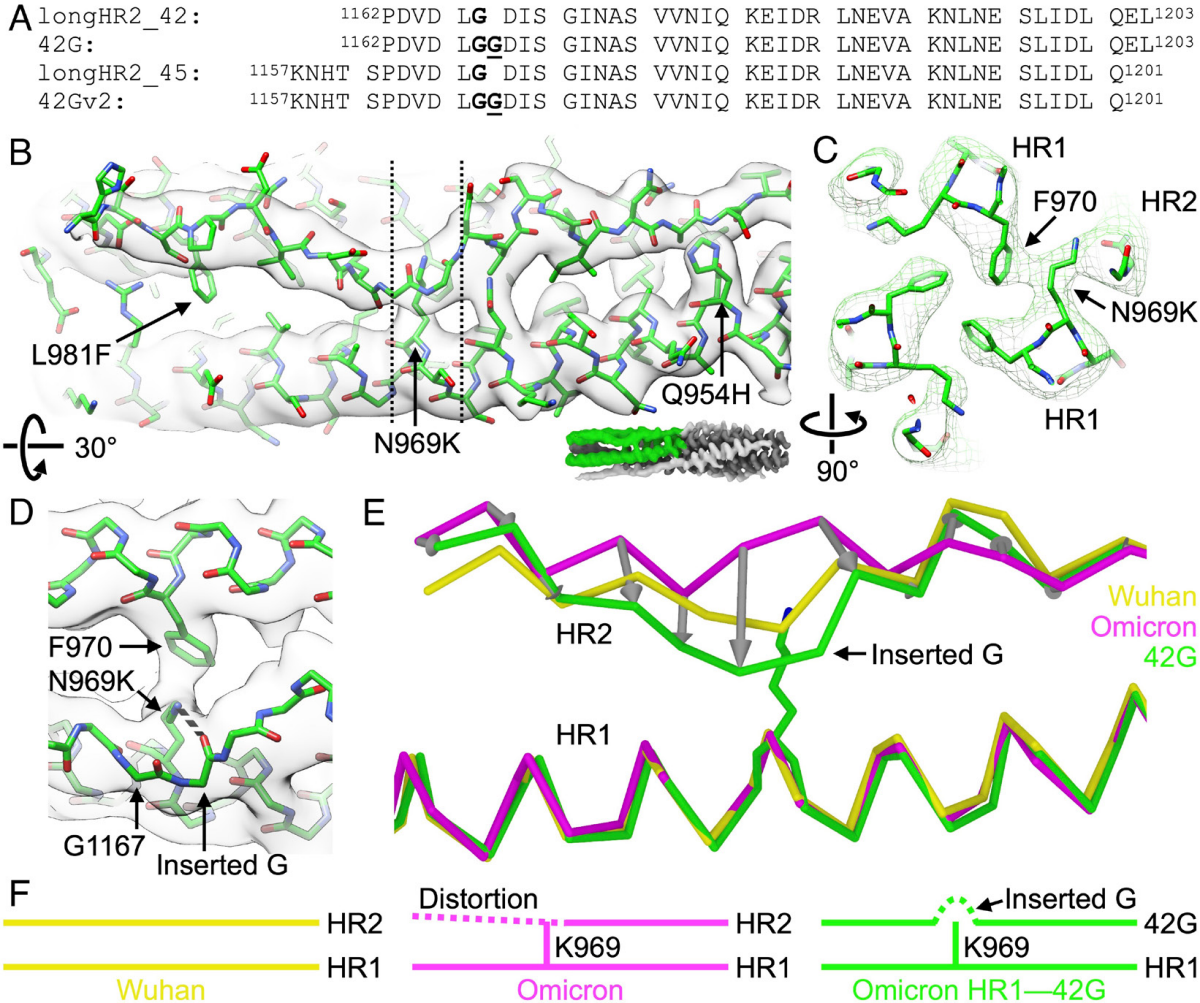
The glycine insertion of the 42G peptide alleviates the HR2 backbone shift induced by the N969K mutation in HR1. (*A*) Primary amino acid sequences of the longHR2_42, 42G, longHR2_45, and 42Gv2 peptides. Residue G1167 is in bold, and the inserted glycine residues is in bold and underlined. (*B* and *C*) Cryo-EM map and structure of the complex of Omicron HR1 and the 42Gv2 peptide (PDB ID 8fa2, EMDB ID 28948, this study). Green: carbon atoms. Blue: nitrogen atoms. Red: oxygen atoms. Only one 42Gv2 protomer and its two neighboring HR1 protomers are shown for clarity, and the displayed region of the bundle is colored green in the lower right inset. The dashed vertical lines indicate the slab that is displayed in panel *C*. (*D*) Close-up view around the mutated N969K residue and its interactions with F970 and the inserted glycine of 42G. The map and structure are rotated 30°, compared to that shown in panel *A*. Backbone atoms of 42G are shown, and the hydrogen bond between the Z nitrogen of N969K and the carbonyl oxygen of the inserted glycine residue is shown as a dashed line. (*E*) Superposition of the Cα ribbons of the postfusion HR1HR2 bundles of the Omicron HR1—42Gv2 complex shown in panel *B*, of the Wuhan strain, and of the Omicron variant. Only one 42Gv2 protomer and one neighboring HR1 protomer are shown for clarity. Gray arrows connecting the Cα atoms of Omicron HR2 backbone and of the 42Gv2 illustrate that the displaced backbone of Omicron is partially restored to the Wuhan strain conformation. (*F*) Graphic summary of the effect of the Omicron N969K mutation on the nearby HR2 region and accommodation of the mutation by the inserted glycine residue in the 42G peptide.

Our structures suggest that the inhibition activity against the Omicron variant can be improved if an inhibitory peptide better accommodates the HR1 N969K mutation than the previous wildtype HR2 peptides used. Since the N969K mutation causes the displacement of the HR2 backbone (Fig. 1*C*), a nearby complementary residue substitution to HR2 that only changes the sidechain is unlikely to restore the local geometry. Upon the inspection of the N696K mutant structure (Fig. 1), we predicted that the insertion of an additional residue in HR2 near the Omicron HR1 K969 residue might better accommodate the K969 mutation, thus promoting a more stable interface between HR1 and HR2. Starting with the previously reported longHR2_42 peptide (13) we chose to insert an additional residue between HR2 residues G1167 and D1168 which are proximal to the Omicron HR1 K969 sidechain (Fig. 2*A*), anticipating that this insertion would restore inhibition activity. We selected glycine due to its flexibility, hydrophilicity, and proximity to the naturally occurring glycine in HR2 (G1167) (peptide referred to as 42G).

To confirm our prediction that 42G better accommodates the Omicron N969K mutation, we determined the cryo-EM structure of the complex of the Omicron HR1 and a slightly longer version of 42G, termed 42Gv2, to an overall resolution of 2.8 Å (Fig. 2*B* and *SI Appendix*, Fig. S1 and Table S1). We used the slightly longer 42Gv2 construct for structure determination since it has the same residue range as the longHR2_45 peptide (13) used for structure determination of the Wuhan, Omicron, and the N969K postfusion bundles (Fig. 1). The structure of the 42Gv2—Omicron HR1 complex maintains the overall six-helix-bundle architecture of the SARS-CoV-2 postfusion bundle and the packing between K969 and F970 (Fig. 2*C*). In addition, the structure features a bulge of the 42Gv2 backbone at the inserted glycine residue (Fig. 2*D*). This bulge results from the deviation of the 42Gv2 backbone around the long sidechain of the lysine residue of the mutated HR1 N969K, promoting closer overall packing with HR1; additionally, the terminal nitrogen of this lysine and the carbonyl oxygen of the inserted G form a hydrogen bond, presumably contributing additional binding energy to the interface (Fig. 2*D*). Additionally, the insertion reduces the overall displacement of HR2 in this region, partially restoring the HR2 backbone to the original position in the Wuhan strain structure (Fig. 2 *E* and *F* and *SI Appendix*, Fig. S2 and Movie S1). The average Cα atom rmsd for HR2 residues 1,162 to 1,170 is reduced from 2.4 Å (between the Wuhan strain HR1HR2 structure and the Omicron HR1HR2 structure) to 1.5 Å (between the Wuhan strain HR1HR2 structure and the Omicron HR1—42Gv2 structure). Our structure of the Omicron HR1—42Gv2 complex shows that the glycine insertion restores the backbone interactions on both sides of the inserted glycine and Omicron HR1 (Fig. 2 *E* and *F* and *SI Appendix*, Fig. S2 and Movie S1). Thus, the N-terminal extension (residues 1,162 to 1,167), previously found to be critical for the peptide’s potent inhibition against infection (13), interacts with HR1 in a similar manner as observed in the Wuhan structure. Moreover, these results suggest that the interaction between the N-terminal extension and HR1 is sequence-specific.

### Omicron-Specific Inhibition by 42G in a Cell–Cell Fusion Assay.

**Fig. 3.**
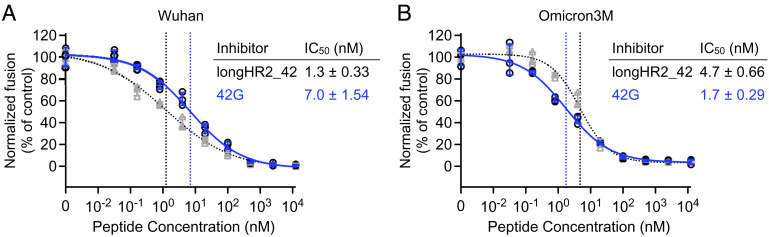
The optimized 42G peptide improves the inhibition of Omicron in a cell-cell membrane fusion assay. Inhibition activities by 42G against Wuhan (*A*) or Omicron3M (*B*) S protein. For 42G, the raw data points are plotted as black circles, while the error bars (SD), fitted curves, and vertical dashed lines at IC_50_ are plotted in blue color. For longHR2_42, the raw data are plotted as gray triangles with gray error bars (SD), while the fitted curves, and vertical dashed lines at IC_50_ are colored black. The data for longHR2_42 in *A* were previously published in ref. 13. Details about the number of repeats, calculation of means, fitting, and calculation of error of the fit are in the *Materials and Methods*.

To test whether the glycine insertion in the 42G peptide restores inhibition activity in a cell–cell membrane fusion assay using S protein and ACE2, we used the Omicron triple mutant (hereafter referred to as “Omicron3M”) that was used in our previous study (14). Omicron3M contains only the three mutations in the HR1 region, but not mutations in other regions of Omicron S. Compared to the original longHR2_42, the 42G peptide has an ~fivefold reduced inhibition activity against fusion with the Wuhan strain (Fig. 3*A*) but has an ~threefold enhanced inhibition activity against fusion with the Omicron3M, recovering the loss of inhibition activity of the original longHR2_42 peptide conferred by Omicron (Fig. 3*B*). At the same time, 42G still inhibits fusion with Wuhan strain S protein, albeit less so than the original longHR2_42 peptide. Thus, insertion of a glycine residue in the peptide enhances its Omicron-specific inhibition activity in our cell–cell fusion assay.

### Omicron-Specific Inhibition by 42G in a VSV-SARS-CoV-2 Chimera Infection Assay.

**Fig. 4.**
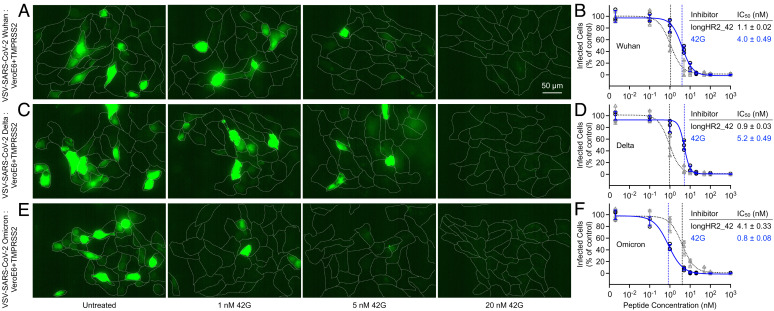
VSV-SARS-CoV-2 chimera infection assay confirms the Omicron-specific inhibition by the 42G peptide. Inhibition of VSV-SARS-CoV-2 Wuhan (*A* and *B*), VSV-SARS-CoV-2 Delta (*C* and *D*), or VSV-SARS-CoV-2 Omicron (*E* and *F*) infection by 42G in VeroE6+TMPRSS2 cells. Virus and peptide were incubated with cells for 1 h, washed, then fixed and imaged 8 h after initiation of infection allowing for one round of infection to occur. Images are maximum intensity projections of 20 µm z-planes taken with 1 µm spacing using a spinning disk-confocal microscopy (*Materials and Methods*). Expression of a soluble eGFP (green) reporter allowed for infected cells to be determined while cell outlines were obtained from WGA-Alexa647 stain applied immediately prior to fixation (*A*, *C*, and *E*). In panels *B* and *D*, and *F*, the plots are colored the same as Fig. 3. The data for longHR2_42 in *B*, *D*, and *F* were previously published in ref. 13. Details about number of repeats, calculation of means, fitting, and calculation of SE are in the *Materials and Methods*.

We next tested the 42G peptide for variant-specific inhibition activity using a VSV-SARS-CoV-2 chimera infection assay, which has been previously shown to strongly recapitulate the entry route of natural SARS-CoV-2 (13, 16, 17). The chimeric virus was produced by replacing the glycoprotein of VSV with the S protein of Wuhan, Delta, or Omicron strains of SARS-CoV-2. The first round of VSV-SARS-CoV-2 infection was examined in VeroE6 cells overexpressing the transmembrane protease, serine 2 (TMPRSS2) (VeroE6+TMPRSS2) by expression of a soluble cytosolic eGFP reporter encoded in the virus 8 h after inoculation. Compared to the original longHR2_42, the 42G peptide displayed an ~fivefold enhanced inhibition activity against Omicron infection (Fig. 4*C*) and ~fourfold to sixfold decreased inhibition activities against Wuhan (Fig. 4*A*) and Delta strains infection (Fig. 4*B*). These results were consistent with the Omicron-specific enhanced inhibitory activity of 42G observed with the cell–cell fusion assay (Fig. 3).

## Discussion

One striking difference between SARS-CoV-2 Omicron and prior variants is a marked increase in mutational load, including within the S protein. New mutations emerged both at antigenic interfaces under heavy selection pressure, such as the RBD, and at more distant but functionally critical sites, such as the S protein fusion core comprised of the HR1 and HR2 regions. These mutations within the fusion core of Omicron lead to major conformational changes of HR2 in the structure of the HR1HR2 postfusion bundle (14) (Fig. 1) and a decreased activity of a potent HR2-based inhibitor against infection (13) (Fig. 4).

Here, we sought to recover the activity of the previously reported inhibitor through a rational, structure-based design. First, we identified the Omicron N969K mutation as the primary cause of the structural change in the Omicron HR1HR2 postfusion bundle, the sidechain of which displaces the HR2 backbone in its N-terminal extended region (Fig. 1*C*). Then, we designed an optimized inhibitory peptide, 42G, to accommodate the long lysine sidechain by insertion of a highly flexible glycine residue in proximity to the lysine sidechain of Omicron HR1 (Fig. 2). The high-resolution structure of a slightly longer version of 42G, 42Gv2, bound to Omicron HR1 shows that the glycine insertion allows the HR2 backbone to bypass the lysine residue in the mutated HR1 N969K and remain closer to the Wuhan strain conformation of HR2 with minimal disruption of backbone geometry (Fig. 2 and *SI Appendix*, Fig. S2 and Movie S1). This glycine insertion allows for the formation of a bulge perpendicular to the bundle (Fig. 2*D*), permitting the HR2 residues on both sides of the mutated lysine residue (HR1 N969K) to adapt a conformation closer to that observed in the Wuhan strain (Fig. 2*E*). This improvement of 42G interactions with Omicron HR1 translates to increased inhibition activity for the Omicron variant as observed in both cell–cell fusion and VSV-SARS-CoV-2 chimera infection assays (Figs. 3 and 4). 42G still inhibits Wuhan and Delta strain infection, albeit less so than the original longHR2_42 peptide which was based on the Wuhan strain sequence.

These observations suggest that sequence-specific interactions between HR2 (1,162 to 1,167) and HR1 (969 to 981) residues account for the improved inhibition activity of 42G against Omicron. Functionally, these interactions may mediate the initial landing of HR2 onto HR1 during the transition of the S protein from the prehairpin intermediate to the postfusion state (13), assuming the zippering of the HR1HR2 bundle begins from the N terminus of HR2.

Given our observation that the HR1 N969K mutation destabilizes the postfusion conformation of S, it is perhaps surprising that all existing Omicron subvariants retain the N969K mutation. Recently, McCallium et al. (18) showed that the Omicron N969K and L981F mutations stabilize the prefusion conformation through interprotomer electrostatic interactions and intraprotomer hydrophobic packing, respectively. However, the L981F mutation is probably not required for tolerating the destabilization of the postfusion S by the N969K mutation, since several Omicron subvariants do not have the L981F mutation. It is unclear if the Omicron Q954H mutation has a functional role, although we previously showed that the hydrogen bond between the HR1 residue 954 and the HR2 residue S1175 is maintained for the Q954H mutation (14).

In this study we used two different cell lines—HEK and Vero cells. The ACE2 expression level is very low in HEK cells (19); hence, their ectopic expression is required for efficient fusion. A previous study with a HEK cell–cell fusion assay (20) suggests that the S protein is activated by host cell proteases. Vero cells, which robustly express ACE2, are infected by SARS-CoV-2 upon proteolytic cleavage of the spike protein mediated by endosomal cathepsins (21). Ectopic expression of TMPRSS2 in Vero cells provides a complementary second proteolytic entry route. By using the above two cell systems, we were able to establish that the mode of HR2 peptide inhibition was the same regardless of the entry route used by the virus.

While we focused our studies on the BA.1 subvariant, we expect that the Omicron-specific enhancement of the inhibition activity of 42G should also apply to other recently emerged Omicron BQ.1, BQ.1.1, BA.4.6, BF.7, BA.2.75.2, and XBB.1.5 subvariants (6, 7) since they all retain the N969K mutation. Our study shows that variant-specific inhibitors can be rationally designed in a rapid manner using structures of the postfusion bundles to examine the resistance caused by certain mutations. This will expand the toolbox for the development of variant-specific inhibitors to combat future variants and subvariants of SARS-CoV-2. We envision efficient delivery of peptide inhibitors to the nasopharyngeal cavities and lungs by inhalation in order to achieve targeted delivery. Thus, we anticipate that our nanomolar peptide inhibitors will be excellent leads for clinical drug development.

## Materials and Methods

### Structure Determination.

The cryo-EM structures of the N969K HR1HR2 postfusion bundle and that of the Omicron HR1—42Gv2 complex were determined following a molecular scaffolding method described previously (14). Briefly, the scaffolded complexes were generated by coexpressing the scaffolded HR1 and Small Ubiquitin-like Modifier (SUMO)-tagged HR2 peptides in *E. coli* BL21(DE3) using auto-inducing lysogeny broth (LB) medium (22), followed by nickel affinity chromatography and size exclusion chromatography (SEC) with a Superose 6 Increase 10/300 GL column in 25 mM Hepes-Na, pH 7.4, 150 mM NaCl, 0.5 mM EDTA, 0.5 mM Tris(2-carboxyethyl)phosphine (TCEP). The sample was concentrated to 20 µM, supplemented with 0.05% Nonidet P-40, and plunge frozen on a Quantifoil 2/1 holey carbon grid using a Vitrobot Mark IV (Thermo Fisher). Movie stacks were recorded using a Titan Krios transmission electron microscope (Thermo Fisher) equipped with a K3 camera (Gatan) using the Serial-EM automation software (23), at a nominal magnification of 130,000× and a pixel size of ~0.3 Å. Each movie stack contained ~40 frames with a total electron dose of ~55 e^−^/Å^2^. The data were processed using a combination of MotionCor2 (24), Gctf (25), EMAN2 (26), cryoSPARC (27), and RELION (28), as described previously (14). More details for data collection and processing are summarized in *SI Appendix*, Fig. S1 and Table S1.

For model building, we first slightly improved the structure of Omicron HR1HR2 (PDB 7tik) by better fitting the sidechain of K969 into the corresponding density map (updated statistics are provided in Table S1, and the PDB entry has also been updated). We used this improved model of Omicron HR1HR2 as the initial template for the N969K and Omicron HR1—42Gv2 structures. The model near the glycine insertion was first built in Coot (29) and then refined by an automated structure refinement protocol with Rosetta (30). The structure was then subjected to real space refinement (global minimization, local grid search, adp) in PHENIX (31). Coot (29) was used for further fitting of sidechains and manual inspection.

### Peptide Synthesis and Characterization.

The 42G peptide was synthesized by GenScript USA Inc. HPLC and liquid chromatography–mass spectrometry profiles are shown in *SI Appendix*, Fig. S3 (provided by the manufacturer).

We performed SEC and size exclusion chromatography coupled with multi-angle light scattering (SEC-MALS) to further characterize the 42G peptide in PBS buffer. Peptide powder was first dissolved in dimethyl sulfoxide (DMSO) to ~5 mg/mL. Subsequently, DMSO was exchanged to PBS buffer by three rounds of dilution and concentration. The dilution factor was ~15 for each round, and the centrifugal concentrator (Merck Millipore Ltd.) had a molecular weight cutoff at 3 kDa. The peptide solution was filtered by 0.22 µm PVDF membrane. SEC and SEC-MALS were performed in PBS buffer using a Superdex 75 10 300 GL column and a wtc-010S5 column (Wyatt Technology Corporation), respectively. The SEC and SEC-MALS profiles are shown in *SI Appendix*, Fig. S3. The concentration of the peptide stock solution was determined by absorption measurement at 205 nm using a Nanodrop instrument (Thermo Fisher).

### HEK Cell–Cell Fusion Assay.

We optimized the cell-cell fusion assay (20) based on the α-complementation of *E. coli* β-galactosidase for comparing the inhibitory activity of different peptides with higher throughput. Suspension culture Expi293F cells (Thermo Fisher, Cat.# A14527), a clonal derivative of Human Embryonic Kidney (HEK) 293 cells were grown to a density of 1 ∼ 2 × 10^6^ cells/mL in FreeStyle 293 expression medium (Thermo Fisher, Cat.# 12338026) supplemented with 0.1 mg/mL penicillin-streptomycin antibiotics. The cells were then pelleted, resuspended in medium without antibiotics to a density of 1 × 10^6^ cells/mL, and allowed to recover at 37 °C for 30 min. One group of cells was then cotransfected using polyethyleneimine (PEI, Sigma) (125 μg PEI/mL cells) with Wuhan strain full-length SARS-CoV2 S protein construct (12.5 μg DNA/mL cells) and the α-fragment of *E. coli* β-galactosidase construct (12.5 μg DNA/mL cells) to generate the S protein-expressing cells. Since HEK cells do not express TMPRSS2, it is likely that S is cleaved by host cell proteases that render S fusogenic upon binding to its receptor ACE2 (20). Using the same amount of PEI, the other group of cells was cotransfected with the full-length ACE2 (12.5 μg DNA/mL cells) construct and the ω-fragment of *E. coli* β-galactosidase construct (12.5 μg DNA/mL cells) to generate the ACE2 receptor-expressing cells. Note that the endogenous ACE2 level is very low in HEK cells (19), requiring ectopic expression for efficient fusion. As a negative control, two additional groups of cells were transfected with either the α-fragment or the ω-fragment of *E. coli* β-galactosidase construct alone. After incubation of the cells in flasks at 37 °C for 24 h, the cells were pelleted. The S-expressing cells were resuspended in FreeStyle 293 expression medium supplemented with different concentrations of peptide (50 μL, 2 × 10^6^ cells/mL), respectively. The ACE2-expressing cells and negative control cells were resuspended in 50 μL fresh FreeStyle 293 expression medium (pH 7.4) to be 2 × 10^6^ cells/mL S-expressing, and ACE2 cells or α-fragment and ω-fragment cells were then mixed in a 96-well plate (Greiner Bio-One) to initiate cell-cell fusion at 37 °C for 2 h. Fusion was arrested by adding 100 μL β-galactosidase substrate from the Gal-Screen reporter system (Invitrogen). The mixture was incubated at 37 °C in the dark for 1 h before recording luminescence using a Tecan Infinite M1000.

### Purification of VSV-SARS-CoV-2 Chimeras.

Recombinant VSV chimeras with glycoprotein G replaced with the SARS-CoV-2 S protein with the sequence of the Wuhan-Hu-1 strain, D614G mutation in the Wuhan-Hu-1 strain, Delta strain, or Omicron strain of SARS-CoV-2 (VSV-SARS-CoV-2) and expressing a soluble eGFP infection reporter were generated as described previously (16, 17, 21). VSV-SARS-CoV-2 was grown by infecting 12 to 18 150 mm dishes of MA104 cells at a multiplicity of infection (MOI) of 0.01. Supernatant was collected at 48 h post-infection. The supernatant was clarified by low-speed centrifugation at 1,000 × g for 10 min at 4 °C. Virus and extracellular particles were pelleted by centrifugation in a Ti45 fixed-angle rotor at 30,000 × g for 2 h at 4 °C. The pellet was resuspended in NTE buffer (100 mM NaCl, 10 mM Tris⋅HCl pH 7.4, 1 mM EDTA) at 4 °C. The resuspended pellet was layered on top of a 15% (v/v) sucrose-NTE solution and centrifuged in an SW55 swinging-bucket rotor at 110,000 × g for 2 h at 4 °C. The virus was resuspended in NTE overnight at 4 °C, then separated on a 15 to 45% (v/v) sucrose-NTE linear gradient by ultracentrifugation in an SW55 swinging-bucket rotor at 150,000 × g for 1.5 h at 4 °C. The predominant band in the lower one-third of the gradient was then extracted by side puncture of the centrifuge tube. Virus was then diluted in NTE and concentrated by ultracentrifugation in a Ti60 fixed-angle rotor at 115,000 × g for 2 h at 4 °C. The VSV-SARS-CoV-2 containing pellet was resuspended overnight in NTE in a volume of 0.5 mL and stored at 4 °C for subsequent experiments.

### VSV-SARS-CoV-2 Infection Assay.

Glass slides (18 mm) were cleaned, overlayed with 3-mm polydimethylsiloxane (PDMS) wells and sterilized as previously described (13, 21). On the day prior to the experiment, VeroE6 cells overexpressing TMPRSS2 (Vero+TMPRSS2) were plated inside the PDMS wells on top of the glass slide; the slide was placed in a six-well plate and grown at 37 °C in the presence of 10% CO_2_ to achieve 70 to 80% confluence on the day of the experiment. Note that ACE2 expression is very high in VeroE6 cells (32). On the day of experiments, medium was removed, virus was diluted into media containing the desired concentration of indicated peptide at a final VSV-SARS-CoV-2 concentration of 0.5 µg/mL viral RNA (equivalent to an MOI of ∼0.5 infectious units [IFU] also determined in Vero+TMPRSS2 cells), and then immediately added to the desired PDMS well in a volume of 10 µL for 1 h at 37 °C in the presence of 10% CO_2_. Liquid was placed outside of the PDMS well to maintain humidity and reduce evaporation. Cells were then washed twice with medium to remove unbound virus and peptide inhibitor; the well were then filled with fresh medium. In all experiments, cells were kept at 37 °C with 10% CO_2_, and the medium was prewarmed to 37 °C. At 8 h post-infection, the medium was removed; cells were stained with 5 µg/mL wheat germ agglutin (WGA)-Alexa647 in PBS for 30 s at room temperature. Cells were then washed twice with PBS, fixed with 4% paraformaldehyde in PBS for 15 min, then washed three times with PBS and imaged using a spinning-disk confocal microscope. Imaging was done with a 40× oil objective and an EMCCD camera with a pixel size of 0.33 µm; volume data was obtained by acquiring 20 consecutive planes spaced 1 µm apart for every field of view (33). Cells were considered infected when they displayed a cytosolic eGFP fluorescence signal with a relative intensity at least 1.4 times that of uninfected cells. Example images are maximum-intensity projections highlighting the cell outline marked with the WGA-Alexa647 membrane label.

### Statistics and Data Analysis.

Data from the human embryonic kidney (HEK) cell-cell membrane fusion and VSV-SARS-CoV-2 infection assays from three independent biological replicates determined for each concentration of inhibitor used. For the HEK cell-cell membrane fusion assay, normalized fusion was calculated as

(Luminescence_(+inhibitor)_ − Luminescence_(α&ω)_)/(Luminescence_(+PBS)_ − Luminescence_(α&ω)_),

where “+inhibitor” or “+PBS” refers to adding inhibitor or PBS to the mixture of the cells expressing the α-fragment of *E. coli* β-galactosidase and S, and the cells expressing the ω-fragment of *E. coli* β-galactosidase and ACE2, and “α&ω” refers to the mixture of the cells expressing the α-fragment only and the cells expressing the ω-fragment only.

The infected cells counted in the VSV-SARS-CoV-2 infection assay were normalized by that of control HR2. After the normalization, the arithmetic means of the three replicates were used to fit the inhibition curves to obtain estimates of the IC_50_ values; the estimates were obtained by nonlinear regression of inhibitor concentration vs. response in GraphPad Prism version 9.1.0 for macOS (GraphPad Software, San Diego, CA, https://www.graphpad.com). The fitted model is Y = Bottom + (Top − Bottom)/(1 + (IC_50_/X)^HillSlope^), where Y is the extent of inhibition, X is the inhibitor concentration, Bottom and Top are the minimal and maximal inhibition. The SE of the IC_50_ estimation was calculated using OriginPro 9.1 (OriginLab Corporation).

### Figure Preparation.

The figures of PDB structures and maps were made using UCSF Chimera (34) and PyMOL (The PyMOL Molecular Graphics System, Version 2.5, Schrödinger, LLC). Chains B, C, and E were chosen to display HR1 and HR2 of the Wuhan and Omicron structures. Chains A, C, and F were chosen to display HR1 and HR2 of the N969K and 42G structures. The data fitting of all inhibition assays was performed and plotted using GraphPad Prism version 9.1.0 for macOS (GraphPad Software, San Diego, CA, https://www.graphpad.com).

## Supplementary Material

Appendix 01 (PDF)

Dataset S01 (XLSX)

Dataset S02 (XLSX)

Movie S1. Video showing rocking views of the superposition of the cryo-EM maps and structures of the HR1HR2 postfusion bundles of the Wuhan strain (yellow, PDB ID 8czi, EMDB ID 27098), of the Omicron variant (magenta, PDB ID 7tik, EMDB ID 25912), of the N969K mutant (cyan, PDB ID 8fa1, EMDB ID 28947, this study), and of the Omicron HR1—42Gv2 complex (green, PDB ID 8fa2, EMDB ID 28948, this study). Structures are shown as lines, HR1 residues 969 and 970 are shown as sticks, and maps are shown as meshes (contour level 5). Blue: nitrogen atoms. Red: oxygen atoms.

## Data Availability

The EM maps and corresponding structures reported here have been deposited in EMDB (Electron Microscopy Data Bank) and PDB with the following accession IDs: HR1HR2 N969K: EMDB 28947 (35), PDB 8fa1 (36); and Omicron HR1—42Gv2: EMDB 28948 (37), PDB 8fa2 (38).
