# Supplementary material for: Structure-based design of a SARS-CoV-2 Omicron-specific inhibitor
Source: Proc Natl Acad Sci U S A. 2023 Mar 20;120(13):e2300360120. doi: 10.1073/pnas.2300360120 (PMC10068829; doi:10.1073/pnas.2300360120)
Supplement: Supplementary file 1 — Appendix 01 (PDF) [file pnas.2300360120.sapp.pdf]

Supporting Information for
Structure-based discovery of a SARS-CoV-2 Omicron-specific inhibitor

Kailu Yang, Chuchu Wang, Alex J. B. Kreutzberger, K. Ian White, Richard A. Pfuetzner, Luis Esquivies, Tomas Kirchhausen, and Axel T. Brunger

*Corresponding author: Axel T. Brunger
Email: brunger@stanford.edu

This PDF file includes:

Figures S1 to S3
Table S1
Video Legend S1

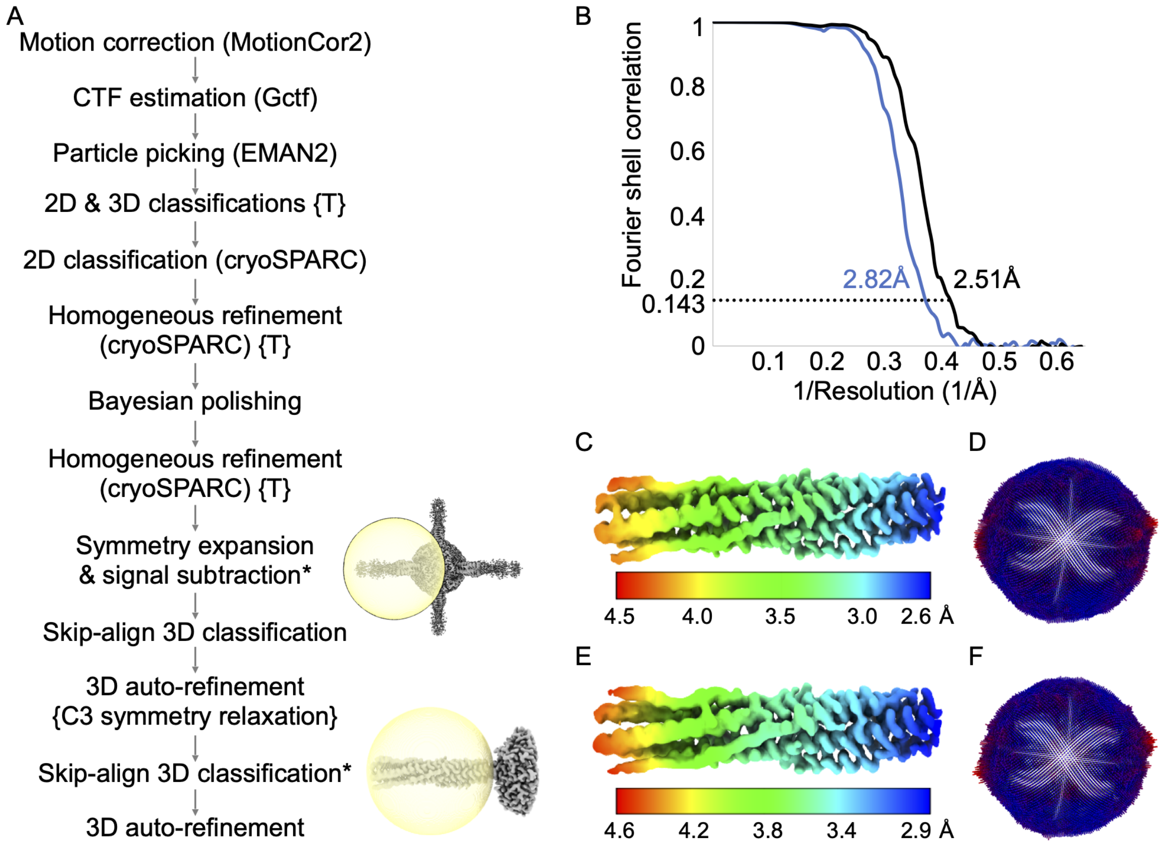

Fig. S1. Cryo-EM structure determination for the N969K and HR1—42Gv2 postfusion bundles. (*A*) Workflow of cryo-EM data processing. RELION was used for each step unless another program is indicated in parenthesis. Symmetry was imposed or relaxed as indicated in curly brackets. The steps that used a manually generated mask (rather than the default spherical mask in RELION or the default dynamic masks in cryoSPARC) are indicated with a star sign and an image showing the manually generated mask (yellow) and an average map (gray). (*B*) Fourier shell correlations (FSC) of the final local refinements with RELION (last 3D auto-refinement). Black: the N969K mutant structure. Blue: Omicron HR1—42Gv2 structure. Note that these FSC calculations were performed using the default spherical mask that covers both the HR1HR2 bundle and part of the scaffold. The final reconstructions of the N969K mutant structure (*C*) and the Omicron HR1—42Gv2 structure (*E*) are colored by local resolutions. Distributions of the particles' orientations in the final reconstructions of the N969K mutant structure (*D*) and the Omicron HR1—42Gv2 structure (*F*) are depicted in the same orientation as the map in (*C*) and (*E*), respectively. The length of each bar is proportional to the number of particles oriented in that direction. The bars are also colored based on the length, with red meaning more particles and blue meaning fewer particles.

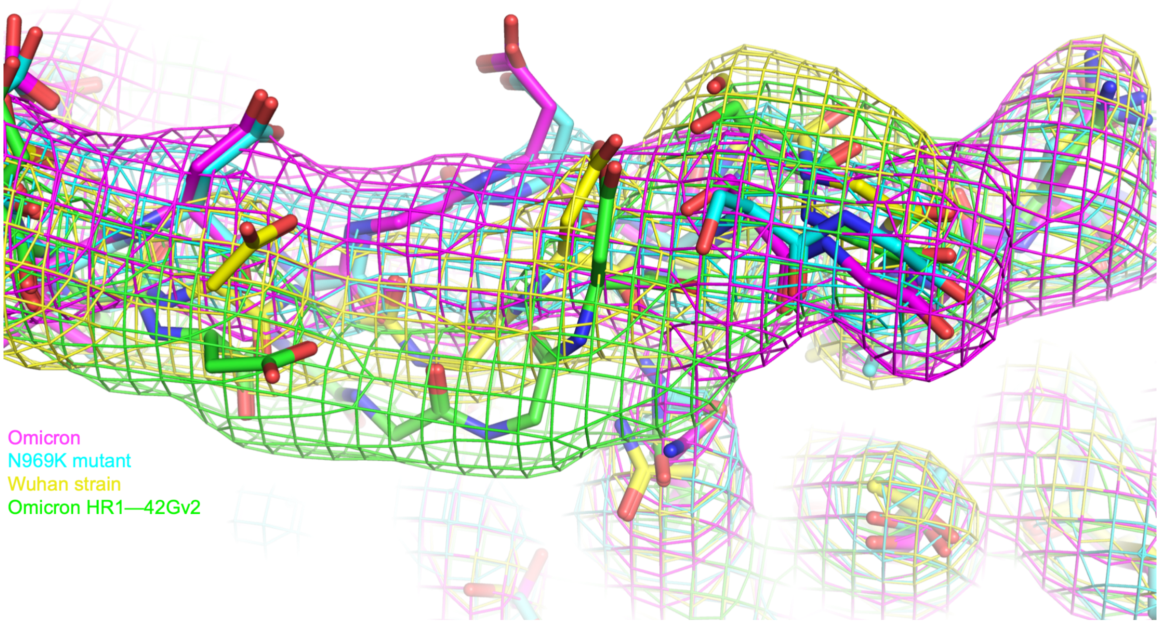

Fig. S2. Superposition of the cryo-EM maps and structures of the HR1HR2 postfusion bundles of the Wuhan strain (yellow, PDB ID 8czi, EMDB ID 27098), of the Omicron variant (magenta, PDB ID 7tik, EMDB ID 25912), of the N969K mutant (cyan, PDB ID 8fa1, EMDB ID 28947, this study), and of the Omicron HR1—42Gv2 complex (green, PDB ID 8fa2, EMDB ID 28948, this study). Structures are shown as lines and maps are shown as meshes (contour level 5). Blue: nitrogen atoms. Red: oxygen atoms. See Supplementary Video S1 for a more complete view.

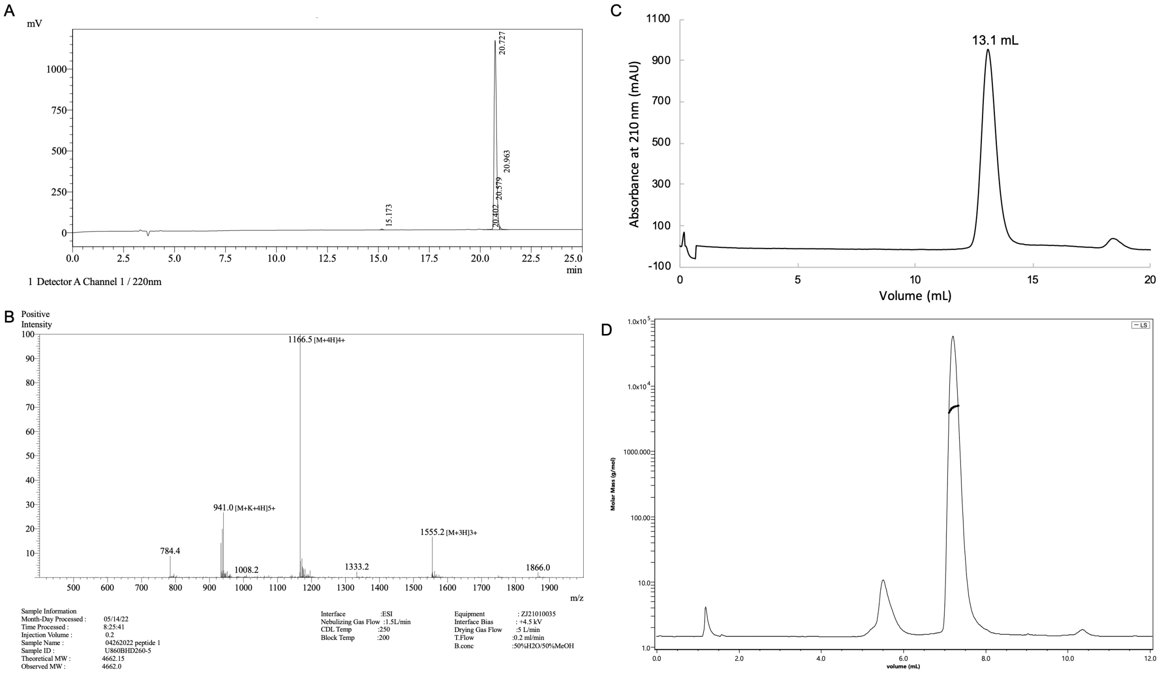

Fig. S3. HPLC (*A*), LC-MS (*B*), SEC (*C*), and SEC-MALS (*D*) profiles of the 42G peptide. The molecular weight measured by SEC-MALS is 4.7 ± 0.09 kDa, the same as the theoretical monomer molecular weight.

Table S1. Cryo-EM data collection, refinement, and model building.

	Omicron	N969K	Omicron HR1—42G
EMDB	25912	28947	28948
PDB	7tik	8fa1	8fa2
Microscope	Titan Krios	Titan Krios	Titan Krios
Voltage (kV)	300	300	300
Camera	Gatan K3	Gatan K3	Gatan K3
Pixel size (Å)	0.65	0.6432	0.653
Exposure time (s)	1.5	1.49	1.012
Number of frames per exposure	40	41	40
Total Dose (e^-/Å^2)	51.7	55	57
Number of movies	19,898	26,382	26,962
Defocus range (μm)	-2 to -0.3	-2 to -0.3	-2 to -0.3
Number of particles	546,372	883,152	547,178
Resolution of final global refinement (0.143 FSC, Å)	1.77	1.77	1.92
Resolution of final local refinement (0.143 FSC, Å)	2.42	2.51	2.82
Bond RMSD (Å)	0.006	0.006	0.006
Angle RMSD (°)	0.724	0.572	0.546
Molprobity score	1.95	1.76	1.36
Clashscore, all atoms	13.36	8.14	6.56
Ramachandran favored (%)	95.41	95.41	98.18
Ramachandran allowed (%)	4.59	4.59	1.82
Ramachandran outliers (%)	0	0	0
Rotamer outliers (%)	0	0	0
Cβ outliers (%)	0	0	0
CaBLAM outliers (%)	0.95	0	0

Video S1. Video showing rocking views of the superposition of the cryo-EM maps and structures of the HR1HR2 postfusion bundles of the Wuhan strain (yellow, PDB ID 8czi, EMDB ID 27098), of the Omicron variant (magenta, PDB ID 7tik, EMDB ID 25912), of the N969K mutant (cyan, PDB ID 8fa1, EMDB ID 28947, this study), and of the Omicron HR1—42Gv2 complex (green, PDB ID 8fa2, EMDB ID 28948, this study). Structures are shown as lines, HR1 residues 969 and 970 are shown as sticks, and maps are shown as meshes (contour level 5). Blue: nitrogen atoms. Red: oxygen atoms.
